# Outcomes of Elderly Patients Undergoing Emergency Surgery for Complicated Colorectal Cancer: A Retrospective Cohort Study

**DOI:** 10.6061/clinics/2019/e1074

**Published:** 2019-08-13

**Authors:** Carlos Augusto Metidieri Menegozzo, Frederico Teixeira-Júnior, Sérgio Dias do Couto-Netto, Octacílio Martins-Júnior, Celso de Oliveira Bernini, Edivaldo Massazo Utiyama

**Affiliations:** Divisao de Cirurgia Geral e Trauma, Hospital das Clinicas HCFMUSP, Faculdade de Medicina, Universidade de Sao Paulo, Sao Paulo, SP, BR

**Keywords:** Colorectal Neoplasms, Colorectal Surgery, Postoperative Complications, Elderly, Emergency Treatment, General Surgery, Surgical Oncology

## Abstract

**OBJECTIVE:**

Colorectal cancer is one of the most frequent types of malignant neoplasms. Age is a risk factor for this disease, with 75% of cases diagnosed in patients older than 65 years. Complications such as obstruction, hemorrhage, and perforation are present in more than one-third of cases and require emergency treatment. We aim to analyze the profile of elderly patients undergoing surgery for complicated colorectal cancer, and to evaluate factors related to worse short-term prognosis.

**METHODS:**

A retrospective analysis of patients who underwent emergency surgical treatment for complicated colorectal cancer was performed. Demographics, clinical, radiological and histological data were collected.

**RESULTS:**

Sixty-seven patients were analyzed. The median age was 72 years, and almost half (46%) of the patients were female. Obstruction was the most prevalent complication at initial presentation (72%). The most common sites of neoplasia were the left and sigmoid colon in 22 patients (32.8%), and the right colon in 17 patients (25.4%). Resection was performed in 88% of cases, followed by primary anastomosis in almost half. The most frequent clinical stages were II (48%) and III (22%). Forty-three patients (65.7%) had some form of postoperative complication. Clavien-Dindo grades 1, 2, and 4, were the most frequent. Complete oncologic resection was observed in 80% of the cases. The thirty-day mortality rate was 10.4%. Advanced age was associated with worse morbidity and mortality.

**CONCLUSION:**

Elderly patients with complicated colorectal cancer undergoing emergency surgery have high morbidity and mortality rates. Advanced age is significantly associated with worse outcomes.

## INTRODUCTION

Colorectal cancer is the third most common malignancy in men, the second most common malignancy in women, and the fourth most significant cause of death from malignant neoplasms in the world 1. In 2012, there were an estimated 1.4 million cases and more than 690.000 deaths. Worldwide mortality trends vary and are increasing in less developed countries with limited resources such as Brazil 2. According to the Brazilian National Institute of Cancer (INCA), in 2016, there were 16.660 and 17.620 estimated new cases in men and women, respectively 3.

Despite screening methods, up to 43% of patients will develop some form of complication including obstruction, perforation or hemorrhage 4-7. Emergency surgery has a direct impact on results. There are higher postoperative complication and mortality rates with emergency surgery than with elective surgery, reaching 33.6-64% and 20-34%, respectively 8-10. Moreover, old age is considered a risk factor for emergency surgery in patients with colorectal cancer 11,12.

There are few reports in the literature specifically evaluating elderly patients with complicated colorectal cancer (CCC). This scarceness of data is even more pronounced when analyzing studies from developing countries, where for various reasons, the colorectal cancer incidence is increasing, and more complicated cases are being managed 2.

The objectives of this study are to analyze the profile of elderly patients treated for CCC and to evaluate factors associated with a worse prognosis in the short term.

## METHODS

This study was approved by the institution's ethics committee and is registered in the Research Registry (www.researchregistry.com) under the number 3886. We conducted a retrospective single-center analysis of patients undergoing surgical treatment for CCC, including all patients 60 years or older who were admitted to the emergency surgical department. Those diagnosed by other services, those who did not undergo emergency surgery, or those whose histopathological analysis did not confirm malignancy were excluded.

The variables collected from medical records included age, gender, tumor site, clinical presentation and staging, surgical treatment, and postoperative follow-up. The World Health Organization defines elderly as patients aged 60 years or older for developing countries. The American Society of Anesthesiology (ASA) score was used to assess patients' comorbidities according to preoperative evaluation findings 14. Postoperative complications were classified according to the Clavien-Dindo classification 15, validated for emergency surgery 16, and divided into early and late (before or after 30 days). The American Joint Committee on Cancer (AJCC) classification was used for clinical staging based on perioperative data 17. We analyzed the mortality rate during the first 30 days of admission or hospital stay.

We classified the surgical procedures into four types: resection and anastomosis, resection and stoma, diverting loop colostomy without resection, and sole biopsy. The histological analysis included the grade of differentiation, surgical margins, and lymph nodes harvested. When resection was not feasible or neoadjuvant (chemotherapy or radiation therapy) was indicated, biopsies were performed.

Statistical analysis was conducted to define variables associated with morbidity and mortality. Chi-squared, Fisher's exact and Mann-Whitney U tests were performed using STATA software (STATACorp. 2007. Stata Statistical Software: Release 10.0. College Station, Texas: Stata Corporation). The normality of the data was evaluated using the Shapiro-Wilk test. The confidence interval was 95%, and *p*- values <0.05 were considered significant.

## RESULTS

Sixty-seven elderly patients were included in this study during a period of four years. Table 1 summarizes the demographic, clinical and pathological data of these patients.

**Figure 1:**
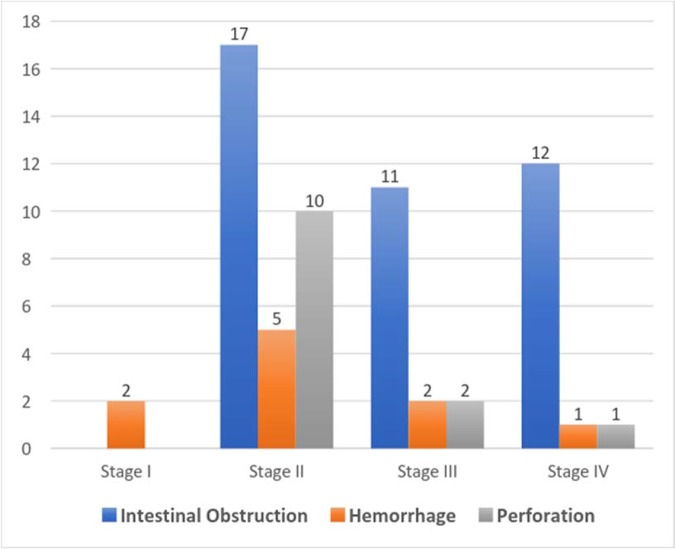
Distribution of clinical presentations according to TNM staging (AJCC) in the studied population.

Figure 1 depicts the clinical presentations associated with TNM staging (AJCC). In one case, only biopsy was performed due to diffuse peritoneal carcinomatosis. Regarding the histopathological analysis, a mucinous component was identified in 8 cases. Seven patients underwent diverting loop colostomy and biopsy, yielding no specimens for margin or lymph node evaluation.

The average total and intensive care unit (ICU) lengths of stay were 18 and 5 days, respectively. Forty-four patients (64.7%) developed a total of 77 postoperative complications (Table 2). Of these, 48% were grades 1 or 2, and 39% were grades 3 or 4. There were 9 cases (13%) of reoperation, 6 of anastomotic leakage, 2 of stoma necrosis, and one of refractory shock. Seven patients (10%) died during hospitalization or within the first 30 days after surgery, of which two underwent reoperation. Septic shock was the cause of death in 5 patients. Table 3 summarizes the relationship between the variables and the incidence of postoperative complications and mortality.

## DISCUSSION

Our results show that elderly patients undergoing emergency surgery for CCC have a high morbidity rate (64.7%) and a considerable mortality risk (10.4%) during the first 30 postoperative days. Approximately 50% of the complications were easily manageable and tended to have low clinical impact. However, a third of them that were grade 3 and 4 complications according to the Clavien-Dindo classification were relevant.

Unlike most of the studies showing a higher prevalence of colorectal neoplasms in male patients 18,19, our work has demonstrated that women composed almost half of the study population. This difference might be explained because the analysis was restricted to patients who underwent surgery in the emergency setting. The same difference has been observed in other studies when analyzing a similar subset of patients 6,18,20,21.

Despite the current recommendations for colorectal cancer screening in Brazil, patients still present with some complications from advanced disease due to late diagnosis 20,22. Our study confirmed similar findings, showing that 28 patients (41%) with CCC presented with clinical stage III or IV. Other studies have also reported advanced stages in patients undergoing emergency surgical treatment 6,7,22.

The main emergencies related to colorectal neoplasia are intestinal obstruction, hemorrhage, and perforation. Intestinal obstruction is the most frequent, in 30.4-84.2% 5,8,9,13,18,20 and was the reason for surgery in 61% of our cases. The occurrences of CCC, advanced stage disease, metastatic disease, and emergency surgery were more frequent among elderly patients than among their younger counterparts 9,12,18,20,23. Moreover, a recent analysis of almost 7000 patients with colorectal cancer identified that advanced age, African-American ethnicity, an increased number of comorbidities, and a more advanced stage of the disease are associated with emergency surgery 6. Access to health services varies according to age, ethnicity and socioeconomic status, increasing admissions for CCC 7 and resulting in worse postoperative outcomes than elective surgery 4,6,20,24. Nonetheless, some studies have suggested that this difference is due to underlying conditions, clinical performance status, smoking, and alcohol abuse, and has no direct relationship to emergency treatment or age 20,23,25.

Surgery with curative intent is feasible in elderly patients undergoing surgery in the emergency setting 26, and the operative decision depends on the clinical presentation, patient's condition and comorbidities. Ideally, the surgeon must comply with the oncological principles of en-block resection, free margins, and adequate lymphadenectomy 27. Alternatively, in emergency presentations, a diverting loop colostomy may be the procedure of choice. Emergency surgery for colorectal cancer results in higher rates of positive margins and inappropriate lymphadenectomies 7. In our series, resection was feasible in 61 (89.7%) cases, with R0 in 58 (95%). In previous studies, emergency surgical resections were performed in 69-90% of cases 9,25, specifically R0 in 55-92% 7,8,20,21. Regarding lymphadenectomy, 79% of our patients had 12 or more lymph nodes harvested. This number ranges from 68.7% to 79.9% in other series 6-8. Adequate lymph node resection results in higher survival rates 6. With respect to adenocarcinoma differentiation, our results are consistent with the literature, showing that moderately differentiated types are more prevalent 22,28.

Traditionally, in emergency presentations, tumors of the ascending colon are managed with resection and primary anastomosis, while Hartmann's procedures are performed for left colon neoplasms. However, primary anastomosis in the latter situation has shown good results in selected cases while avoiding stomas, which are associated with closure rates of less than 20% 5,8,29,30. Santos et al. published an analysis of 107 adult patients who underwent surgery for CCC. Of those, 52 were older than 60 years. Hartmann's procedure was performed in most cases (85%), while only 10% of patients underwent primary anastomosis. The mortality rate was 34%, and septic shock was the leading cause of death. When analyzing the subset of patients older than 60 years, almost half died 10. In our report, despite inclusion of older patients and an approximately 50% rate of primary anastomosis, the mortality rate was lower. There were nine cases of anastomotic leakage, including 12% in right colectomies and 20% in left colon resections.

The incidence of postoperative complications in our series was 65.7%. While the majority were grades 1 and 2 (low grade), in 33% of patients, they were classified as grades 3 or 4. The same morbidity severity has been reported in 10.3-47.9% of patients 9,13,21. When comparing the 10% mortality rate observed in our series, others show similar results 5,18,20. Higher rates of 18-35.7% 4,19,26,31 have been reported, further highlighting the poor outcomes related to an emergency presentation of CCC in older patients (Table 4). The association between the ICU length of stay and incidence of complications is rather straightforward. However, in this study, because most complications were low grade, our findings highlight the clinical impact of any complication in the population analyzed (median ICU stay in days: 9 *vs*. 3, *p*=0.001), regardless of the severity. To further investigate this possibility, we conducted a subgroup analysis comparing the ICU length of stay between patients with low- and high-grade (Clavien-Dindo >3) complications and found no difference (5 *vs*. 8, *p*=0.055). This finding may suggest that even less severe complications may result in a relevant clinical impact in the elderly population.

Ihedioha et al. examined 358 patients older than 80 years, of whom 99 underwent emergency surgery for colorectal cancer. The complication and 30-day mortality rates were 60.9% and 28.6%, respectively. However, more than 65% of the patients had ASA scores of 3 or 4, highlighting the impact of comorbidities on prognosis 21. Other studies have also correlated higher ASA scores with mortality 13,23,32.

Our study strengthens the observation that advanced age is associated with higher morbidity and mortality even among an elderly population. Others have suggested that the incidence of postoperative complications is elevated in the elderly population and tends to increase with age and the stage of the disease 12,25, also resulting in higher mortality 4,26,28.

Patients admitted with an emergent condition are significantly weaker, exhibit worse clinical performance status and more comorbidities 4. Thus, more effective screening strategies may improve early detection, avoid complications from advanced disease, and yield better outcomes 4,6,7,26,31.

In our study, the lack of data regarding survival or recurrence during follow-up did not allow the assessment of further endpoints. Data show that survival rates beyond 30 days are significantly impaired in this population 31. Furthermore, a recent review including approximately 65,000 patients undergoing emergency surgery for colorectal cancer identified greater 30- and 90-day mortality rates, more readmissions, and a lower 5-year survival rate than in those undergoing elective surgery 7. Yang et al., when considering only patients older than 75 years, showed that emergency surgery, advanced clinical stage, and disease recurrence were related to worse 5-year survival 23.

### Limitations

The retrospective nature of the analysis limits our study. The data presented should be carefully interpreted due to the small size of the cohort. It is possible that, for the same reason, we could not detect an association between the other variables and poor outcomes. This relationship might be observed in larger studies.

Moreover, comorbidities were assessed by the ASA score, which is vulnerable to subjective analysis by different anesthesiologists. Such characteristics may have influenced the results 33. Lastly, we could not gather data on the operative time or performance status scores, which are factors that could impact postoperative morbidity.

## CONCLUSION

Emergency presentations of colorectal cancer in the elderly are frequent, exhibiting higher morbidity and mortality rates than elective admissions. Hence, it is important to recognize such challenging situations as a clinical disparity among nonemergent scenarios. The impact of advanced age on the poor outcomes of this population must be emphasized. This information may improve clinicians' relationships with patients and families, prognostication and management decisions. Despite the critical situation, adequate oncological principles should be pursued, and the importance of that technical aspect must be stressed. This clinical situation in elderly patients needs more investigation with further prospective studies.

## AUTHOR CONTRIBUTIONS

Menegozzo CAM was responsible for the study design, data collection, literature review and manuscript writing. Coutto-Netto SD was responsible for the data collection and manuscript writing. Teixeira-Júnior F was responsible for the study design and literature review. Martins-Júnior O was responsible for the study design and data collection. Bernini CO and Utiyama EM were responsible for the manuscript critical final review.

## Figures and Tables

**Table 1:** Demographic, clinical and pathological data of 67 elderly patients with CCC.

	N (%)
Age, years (median, range)	72 (62-97)
Gender	
Male	36 (54%)
Female	31 (46%)
Ethnicity	
White	55 (82%)
African American and Asian	12 (18%)
ASA score	
2	52 (78%)
3	11 (16%)
4	4 (6%)
Comorbidities	
Arterial hypertension	27 (37.9%)
Dyslipidemia	12 (17.9%)
Diabetes Mellitus	8 (12.6%)
Chronic obstructive pulmonary disease	4 (6.3%)
Coronary insufficiency	4 (6.3%)
Vasculopathy	3 (4.2%)
Chagas disease	3 (4.2%)
Renal insufficiency	1 (2.1%)
Other	5 (8.4%)
Clinical Presentation	
Obstruction	48 (72%)
Perforation	10 (15%)
Hemorrhage	9 (13%)
Primary location of neoplasm	
Right colon	17 (25.4%)
Transverse colon	4 (6%)
Left colon	11 (16.4%)
Sigmoid colon	22 (32.8%)
Rectum	13 (19.4%)
Surgical procedure	
Resection + primary anastomosis	30 (45%)
Resection + terminal stoma	29 (43%)
Loop colostomy	7 (10%)
Biopsy	1 (2%)
Grade	
Well-differentiated	9 (13%)
Moderately differentiated	54 (81%)
Poorly differentiated	4 (6%)

**Table 2:** Postoperative complications (Clavien-Dindo 1-5) of 44 patients (n=77).

	N (%)	Clavien-Dindo
Early		
Septic shock	12 (15.6%)	4a, 4b and 5
Anastomotic leak	9 (11.7%)	2, 3b and 5
Acute renal failure	8 (10.4%)	4a
Surgical site dehiscence or infection	11 (14.3%)	2 and 3b
Pneumonia	4 (5.2%)	2
Intrabdominal abscess	4 (5.2%	3a
Urinary tract infection	4 (5.2%)	2
Delirium	5 (6.5%)	2
Venous thromboembolism	1 (1%)	2
Late	6	
Hernia	4	3b
Venous thromboembolism	2	2

**Table 3:** Distribution and statistical comparison of variables and their associations with morbidity and mortality.

Variable	Category / Measures	Morbidity (frequency)		*p* value	30-day mortality (frequency)		*p* value	
		Absent	Present		Absent	Present		
Age	N	23	44	0.003	60	7	
	Range	65-83	62-97		62-97	73-88	0.009
	Median	70	77		71	80	
	Average	70.3	75.9		73.2	80.4	
	Standard dev.	4.3	7.7		7.1	5.2	
Gender	Female	10 (43.5%)	21 (47.7%)	0.740**	26 (43.3%)	5 (71.4%)	0.236*
	Male	13 (56.5%)	23 (52.3%)		34 (56.7%)	2 (28.6%)	
ICU admission	No	9 (45%)	11 (25.6%)	0.123**	18 (32.1%)	2 (28.6%)	0.999*
	Yes	11 (55%)	32 (74.4%)		38 (67.9%)	5 (71.4%)	
ICU length of stay (days)	N	11	32		38	5	
	Range	1-7	1-50	0.001	1-50	3-18	0.073
	Median	2	7		5	9	
	Average	2.8	9.0		7.0	10.6	
	Standard dev.	1.9	9.4		8.8	6.3	

*p*-value by Mann-Whitney U Test.

* *p*-value by Fisher’s exact Test.

** *p*-value by Chi-square Test.

**Table 4:** Morbidity and mortality of elderly patients undergoing emergency surgical treatment.

	Age (years)	Number of patients	Morbidity	Mortality at 30 days
Basili et al., 2008	≥75	20	unspecified	6 (30%)
Kesisoglou et al., 2010	≥70	24	16 (66.6%)	7 (29.2%)
Ihedioha et al., 2013	≥80	98	64 (60.9%)	28 (28.6%)
Santos et al., 2014	≥60	52	unspecified	25 (48%)
Bouassida et al., 2015	≥70	42	unspecified	15 (35.7%)
Menegozzo et al. (current study)	≥60	67	44 (65.7%)	7 (10.2%)
